# Determinants of insecticide treated nets use among youth corp members in Edo State, Nigeria

**DOI:** 10.1186/1471-2458-11-728

**Published:** 2011-09-25

**Authors:** Olorunfemi E Amoran, Idowu O Senbanjo, Chuks E Asagwara

**Affiliations:** 1 Department of Community Medicine and Primary Care, College of Health Sciences, Olabisi Onabanjo University Teaching Hospital, Sagamu, Nigeria; 2 Department of Paediatrics, College of Health Sciences, Lagos State University Teaching Hospital, Lagos, Nigeria; 3 Department of Community Medicine, College of Health Sciences, Igbinedion University Okada, Nigeria

**Keywords:** ITN use, Nigerian Youths, Adoption of ITN, Malaria Prevention

## Abstract

**Background:**

The Africa Malaria Report shows that many countries are quite far from reaching the universal coverage targets of 80% coverage by 2010 and maintain it at this level. This paper examines ITN use and the factors associated with its adoption among the youths in Nigeria. This information will help in the design of effective methods of providing and distributing the nets in order to enhance its adoption and maximize the public health benefits of ITNs.

**Methods:**

This cross-sectional survey was carried out in 2006 among university leavers serving compulsory national service (youth corpers) using total sampling technique. The study was conducted using a self-administered questionnaire.

**Results:**

A total of 656 youth corp members were interviewed. Only 23.8% of these youths ever use ITN while 4.3% currently use ITN before reporting in camp. A significant proportion of the youths acquired information on ITN from Mass Media (p = 0.0001). Other statistically significant factors that encourage the use of ITN include inexpensive market price of ITN (p = 0.0001), frequency of Malaria infestation (p = 0.019) and perceived malaria preventive action of ITN ( p = 0.000).

Following logistic regression analysis, perceived effective malaria preventive action of ITN [OR = 29.3, C.I = 17.17-50.0] and high frequency of Malaria infestation [OR = 1.55, C.I = 0.97-2.47] were predictors of ITN use.

**Conclusion:**

The study shows that the use of ITN for the prevention of Malaria is low among these Nigerian youths. The major factors determining the adoption of ITN among the youths were perceived effective Malaria prevention action of ITN and high frequency of Malaria attack. These factors should be considered in the design of sustainable and effective locally relevant strategies for scale-up adoption of ITNs among a youthful African population.

## Background

The prevalence of Malaria is on the increase globally with sub-Saharan African being the worst hit. There are about 300-500 million clinical cases per year with 80% occurring in Africa. It is responsible for 1 million deaths per year, majorly due to P.falciparum and 90% of which are in Africa [1,2]. Malaria kills about one million people and causes up to 250 million clinical episodes every year [3]. Most of the deaths occur in children under five living in sub-Saharan Africa (SSA), where at least 20% of child deaths are due to malaria [4]. In Nigeria, 1 in 5 children suffers from one episode of severe malaria before the age of 5 years, more than one-third of paediatric admission and one-third of hospital deaths are due to malaria infection [5-7].

The two major measures for malaria control have been prompt and effective treatment and prevention. Bed nets have been used for protection against insect bites long before the discovery that mosquitoes transmitted malaria [8]. In the last decade, there has been a renewed interest in bed nets, and especially insecticide-treated nets (ITN) [4,9]. The Africa Malaria Report shows that many countries are quite far from reaching the target of 60% ITNs coverage in sub-Saharan African countries by the year 2005, which was set in Abuja by the African Heads of State for the provision of ITNs to children under five and to pregnant women [10]. However the 2008 World Malaria Report recommended universal coverage targets of 80% by 2010 and its maintenance at this level. This shift from targeted population to universal coverage i.e. whole population emphasises the current shift of emphasis in control from children and pregnant women alone to whole population in the use of Insecticide-treated nets (ITN) and long-lasting insecticidal treated nets (LLIN) as important means of malaria prevention.

Several studies have demonstrated the efficacy of ITNs [11-19]. The use of ITNs in areas with stable malaria reduced the incidence of uncomplicated episodes by 50% compared to areas where nets were not used, and 39% compared to areas were the nets were untreated. ITNs also impacted on severe malaria, parasite prevalence, high parasitaemia, splenomegaly and improvement in haemoglobin levels of children. This overwhelming evidence of the efficacy of utilization of ITN was the basis of its adoption as one of the four global Roll Back Malaria (RBM) strategies for malaria control. Although there is consensus regarding their importance, there is uncertainty as to which delivery strategies are optimal for dispensing these life saving interventions.

Malaria is a major public health problem in Nigeria and through malaria prevention; ITNs will reduce the need for treatment and the pressure on health services [5,20]. ITN use is one of the major malaria control policy prevention method in Nigeria and the government wishes to scale-up the use of ITNs. Therefore one of the urgent challenges that are facing many sub-Saharan African countries like Nigeria is how to achieve widespread distribution and use of insecticide-treated nets (ITNs) for the control of malaria.

Most studies on ITN use have focused on the use among pregnant women. However this study was carried out to describe factors that determine its adoption among the youths in line with current global emphasis and in view of the fact that they are the future caregivers. The determination of factors that will assure high coverage with the ITNs remains a topical issue in Nigeria and in many sub-Saharan African countries. This study will provide insight into how to develop health promotional goals and activities in order to enhance the use of ITN. This information will help in the design of effective methods of providing and distributing these nets in order to maximize the public health benefits of ITNs and to guide towards nationwide adoption of the strategy especially among the youth who are the future Caregivers.

## Methods

### The Study Area

Edo State was formed on August 27, 1991 when Bendel State was split into Edo and Delta States with Benin City as capital, the population of the entire state is approximately 4 million. It is made up of three major ethnic groups; namely the Binis, Esan and Afemai. However the State has a high presence of residents from across the country and the world because of its cosmopolitan tendencies. The main ethnic groups in Edo State are: Edos, Afemais, Esans, Owans and Akoko Edos. Art, as a form of communication, has been greatly explored, especially for recording memorable events in the life of the various communities. Effigies of Obas, heroes and heroines were molded for posterity. With an estimated population of 3,218, 332 made up of 1,640,461 males and 1, 577, 871 females and a growth rate of 2.7% per annum (NPC, 2006), as well as a total landmass of 19,187 square kilometers, the state has a population density of about 168 persons per square kilometers.

The State has a land mass of 19,794 km square. Lying on 05 44 N and 07 34 N latitudes, 05 4 E and 06 45 E longitudes. Edo State is low lying except towards the north axis where the Northern and Esan plateaus range from 183 metres of the Kukuruku Hills and 672 metres of the Somorika Hills. It is so located that it forms the nucleus of the Niger Delta region. It is bordered by Kogi state to the North and Delta State to the East and South, Ekiti and Ondo States to the West. The climate is typically tropical with two major seasons - the wet (Rainy) and the dry (harmattan) seasons. The wet season lasts from April to November and the Dry Season December to March.

### The Study Population

The youth corpers are the newly graduating students of the various tertiary institutions in Nigeria. They must be below 30 years of age. These university leavers serving compulsory national service must also be of Nigerian origin and completed either a higher diploma or a degree programme in the various discipline in a recognized tertiary institution.

The Corpers are engaged in obligatory national service in two batches every November and February yearly. They are usually engaged in all works of life in the community such as school teachers, medical doctors, engineers in construction companies, accountants etc for the one year mandatory service period. The total number of the corpers in this batch serving in Edo State was 1,500 corpers. About equal number was sent to all the 36 states of the federation including the federal capital territory Abuja. Majority of these youth corps members are Christians, Muslims, and Traditional worshipers.

### Study Design

A cross-sectional survey to determine the prevalence of insecticide treated nets and factors associated with its use among Nigerian youths was carried out in the state NYSC camp in Okada, Edo state among the first batch in February 2006. Total sample of all the consenting Corpers selected to serve in the State was recruited into the study.

### Sampling Procedure

Total sample of all consenting youth Corp members serving in Edo State in February 2006 was done. This was used to obtain a representative sample of the youths in Nigeria.

### Data Collection

#### Pre-testing

The tool is a set of pre-coded questionnaires with open and closed ended questions. The questionnaire was pre-tested among the previous batch of corpers working at the Igbinedion University teaching hospital Okada in Edo State. This community was chosen because of its similarity to the study population. Ambiguous questions were properly rephrased for proper understanding. The experience gathered in the pre-testing was used in organizing the study proper.

#### The questionnaire

The study was conducted using a self-administered questionnaire which was divided into three parts to collect the relevant information. Section one contains the socio demographic data of the respondents while section two sought information on various means of Malaria prevention methods. The final section was the assessment of ITN use by the participants. Current use of ITN was ascertained by asking about the description of use, time, and method of treatment of ITN prior to camping because it was not appropriate to ask for use the night before since it was not provided in camp. The description of this questionnaire is shown in Additional file 1 below.

Permission of the State Director of the NYSC and the state Camp Director was obtained before the commencement of the study. Informed consent was also obtained from the participants before they were enrolled in the study.

### Data Entry and Analysis

Data generated in the study were cleaned for errors and then entered into Computer using SPSS 10 statistical software. Quantitative variables were summarized using means and standard deviations. Chi squared was used to determine association between any two variables. Logistic regression analysis was done to determine factors associated with ITN use among the youths and also to remove the effect of confounding variables. All the variables which were significant in the bivariable analysis with a p < 0.08 were fed into the model. Odd ratios were adjusted and p values of < 0.05 were taken as significant for the study.

#### Ethical clearance

was obtained from the Igbinedion University Teaching Hospital Ethics Board. Confidentiality on candidate's information was maintained. Permission of the State National Youth Service Corps was also obtained before the commencement of the study. Informed consent was also obtained from the parents of the participants before they were enrolled in the study.

## Results

A total of 656 youth corpers were interviewed out of 1,500 youth corpers in camp. The response rate was 43.7% (656 responses). More than half 57.8% of them were males and 42.2% were females. The socio-demographic characteristics of these respondents are shown in Table 1.

**Table 1:** Itn ever use and Socio-dermographic Characteristics

	Total No (%)	ITN Ever Users No (%)	Unadjusted OR [95% C.I]	P value
**Age**				
**18-24 yrs**	106 (16.2)	22 [20.8]	0.81 [0.47-1.39]	0.253
**>24 yrs**	550 (83.8)	134 [24.4]	1.00	
**Sex**				
**Male**	379 [57.8]	86 [22.7]	1.00	0.25
**Female**	277 [42.2]	70 [25.3]	1.15 [0.79-1.68]	
**Professional Discipline**				
**Arts**	137 [20.9]	26 [19.0]	0.87 [0.53-1.42]	0.263
**Basic Sciences**	218 [33.2]	61 [28.0]	1.40 [0.95-2.07]	
**Social Sciences**	279 [42.5]	64 [22.9]	0.70 [0.43-1.15]	
**Medical Sciences**	22 [3.4]	5 [22.7]	0.94 [0.3-2.77]	
**Location of Domicile**				
**South-West**	296 [45.1]	69 [23.3]	0.95 [0.65-1.39]	0.247
**South-East**	140 [21.3]	33 [23.6]	0.99 [0.62-1.56]	
**South-South**	73 [11.1]	14 [19.2]	0.74 [0.38-1.41]	
**North-West**	31 [4.7]	6 [19.4]	0.76 [0.27-1.99]	
**North--East**	35 [5.3]	11 [31.4]	1.50 [0.67-3.30]	
**North-Central**	81 [12.3]	23 [28.4]	1.32 [0.76-2.28]	

### Socio-demographic characteristics of the youth corpers

The mean age of the respondents was 27.4 ± 7.4 years. Only 16.2% of the corpers were less than 24 years and 83.8% were aged between 25-30 yrs. About half [45.1%] of the corpers live in south western part of Nigeria, 21.3% in south eastern part, 11.1% in south-south, 4.7% in the north-west, 5.3% in the north-east and 12.3% in the north central part of the country. Majority of the respondents (80.5%) were Christian, 19.1% were Muslims and the rest were traditional worshippers. Precisely 2.9% of the youth corpers had a degree in Medical sciences, 42.5% had a degree in social sciences, 33.2% in basic science and 20.9% had a degree in Arts.

### The Knowledge of Malaria Prevention and ITN use

Majority (85.5%) of the Corpers could correctly identify the organism responsible for the cause of Malaria. Major methods of Malaria prevention employed by the youth include drugs (17.1%), Screening nets (37.3%), Insecticides spray (30.2%), Environmental care (30.5%), Protective clothing (10.1%) and other methods such as the use of mosquito traps, herbs and spiritual means (19.7%) However all (100%) have heard about ITN before but 89.6% have seen it before.

Only 23.8% of the youths had ever used ITN before but 4.3% of the youths currently use it before reporting to camp. Most (38.4%) of the youths had information about ITN majorly from the mass media, 10.8% had information from friends and relatives, 8.1% from health facilities. 7.2% from schools and 35.5% from other sources such as library, religions groups etc. Over half [67.8%] of the youths believe ITN is affordable and only 32.2% believe it is expensive. Majority (82.6%) of ITN ever users believe that it is a very effective means of Malaria prevention.

### Determinants of ITN use

The overall prevalence of effective use of ITN was 4.3%. There was no statistically significant association between ever use of ITN and age (p = 0.253), sex (p = 0.25), location of domicile (p = 0.247), and professional discipline (p = 0.263).

There was a statistically significant difference between the source of acquisition of information about ITN (p = 0.001), the most effective source of information was the mass media as shown in table 2 below. Other statistically significant factors associated with use include expensive market price of ITN (p = 0.001), frequently of Malaria infestation (p = 0.001).

**Table 2:** Determinants of itn use

	Total No (%)	Ever Users No (%)	Unadjusted OR [95% C.I]	P value	Adjusted OR [95% C.I]	P value
**ITN Malaria Preventive Effect**						
**Effective**	222 [33.8]	135 [86.5]	30.52 [17.76-52.89]	0.0001	29.30 [17.17-50.01]	0.0001
**Non-effective**	434 [66.2]	21 [13.5]	1.00		1.00	
**ITN Price**						
**Expensive**	211 [32.2]	66 [42.3]	1.80 [1.22-2.65]	0.001	1.078 [0.78-1.49]	0.65
**Not-expensive**	445 [67.8]	90 [57.7]	1.00		1.00	
**ITN Information Source**						
**Media**	252 [38.4]	182 [36.4]	1.00		1.00	0.37
**Other Sources**	404 [61.6]	86 [21.3]	0.70 [0.48-1.03]	0.05	0.917 [0.76-1.11]	
**Frequency of Malaria Infection**						
**Greater than once in 6 months**	365 [55.6]	75 [48.1]	1.00	0.019	1.00	0.06
**Once in 6 months or less**	291 [44.4]	81 [51.9]	1.49 [1.02-2.17]		1.548 [0.97-2.47]	

Table 2 shows the adjusted odds ratio for ITN use among the study population. Perceived effective malaria preventive action of ITN (OR = 29.30, C.I = 17.17-50.01) and High frequency of Malaria infestation (OR = 1.55, C.I = 0.97-2.47) increased the probability of use of ITN after controlling for the effect of confounders.

## Discussion

The study shows that only 4.3% of the Nigerian youths currently use ITN while 23.4% have ever used it. This shows that majority of this Nigerian Population are not using ITN regularly despite a lot of emphasis being placed on its use through the roll back malaria programme. The results of this study are comparable to several surveys in Africa, where ITN use varies from 5% to 70% depending on the population studied. Its use is usually high among children and pregnant women [21-23]

The most important method of malaria prevention among the Nigerian youths is screening nets followed by insecticide sprays and environmental engineering. The youths were knowledgeable about the mode of transmission of malaria and the benefits of using malaria preventive methods such as nets and insecticides. This is similar to several other studies among the general population where the commonest mode of malaria prevention was bed nets followed by insecticides sprays and mosquito coils [24,25].

The north-eastern part of the country where there is high malaria infestation which is a measure of perception of risk also shows the highest prevalence of ITNs use. This study shows that malaria infestation is a significant predictor of ITN use. This finding has been confirmed by several other studies that perception of risk of infection by malaria parasite to be a significant predictor of ITN use [6,26-28]

It is not unexpected to find that majority of those who have never used ITN do not believe in its effectiveness. Local knowledge and practice is highly relevant for social marketing strategies of ITNs [7,29]. This has been found to be effective tool in the design of strategies which will encourage the adoption and use of ITNs [30,31]. This indicates that programs that will be geared towards increasing the knowledge and awareness of ITN effectiveness against Malaria prevention should be introduced among the youths.

The most effective source of information among the youths studied was the mass media; the need to educate individuals about malaria and to implement net impregnation services is indicated in this study. This could be done using mass media. Well constructed information on malaria could easily be disseminated to a large number of people in a short time. Health education on mass media should include control of major endemic diseases such as malaria. This will go a long way in the control of these diseases at the community level in developing countries [32,9-18]

About one-third of the sample population believed that the price of acquiring ITNs is expensive. This is similar to findings in several studies which have suggested that the widespread adoption of ITNs will therefore probably require a subsidy [33,34]

Given the cross-sectional nature of the results, interpretation of study results is restricted. A major limitation is the high non response rate reported in this study. This is however expected among these youth corp members in camp except when mandated by the authority to participate in a study. The study might also have been faced with recall bias; however the findings might represent the actual situation since we dealt with educated youths and such experience as ITN use may be very difficult to forget. Future research with a longitudinal approach would be valuable. However, our analyses identified significant relations relevant for social marketing among youths in an African population.

## Conclusion

The use ITN in Nigeria for the prevention of Malaria was low. High malaria infestation which is a measure of perception of risk shows high prevalence of ITNs use. This shows that perception of risk is highly relevant for social marketing strategies of ITNs and the widespread adoption of ITNs. These factors should be considered in the design of sustainable and effective locally relevant strategies for scaled-up adoption of ITNs.

## Competing interests

The authors declare that they have no competing interests.

## Authors' contributions

IOS participated in the study design and conducted data collection. OEA conceived the study theme, participated in the study design, supervised data collection and prepared the final manuscript. CEA was involved in Data collection and analysis. ALL authors read and approved the final manuscript.

## Pre-publication history

The pre-publication history for this paper can be accessed here:

http://www.biomedcentral.com/1471-2458/11/728/prepub

## Supplementary Material

Additional file 1 **Survey Questionnaire**. This file contains the questionnaire used for the survey. Click here for file
