# Supplementary material for: Determinants of insecticide treated nets use among youth corp members in Edo State, Nigeria
Source: BMC Public Health. 2011 Sep 25;11:728. doi: 10.1186/1471-2458-11-728 (PMC3191373; doi:10.1186/1471-2458-11-728)
Supplement: Additional file 1 — Survey Questionnaire. This file contains the questionnaire used for the survey. [file 1471-2458-11-728-S1.DOC]

QUESTIONNAIRE SURVEY ON ADOPTION AND USE OF ITN

We are conducting a survey to learn about Insecticide treated Net (ITN) and Malaria infection. You have been chosen to participate in the study.

We want you to assure you that all of your answers will be kept strictly secret. We will not keep a record of your name or address. There are no right or wrong answers. Some of the answers may be difficult to discuss, but many people have found it useful to have the opportunity to talk.

Please tick the appropriate answer where necessary.

Thank you.

SECTION A

1. Age (in years) -----------------------------------

2. Sex

1. Male

2. female

3. Which town do you live? (Pls specify)--------------------------------------------------

4. Marital status

1. Single, Never married

2. Married

3. Seperated/Divourced

4. Cohabiting

5. Widowed

5. Family type

1. Monogamy

2. Polygamy

6. Tribe

1. Hausa

2. Yoruba

3. Igbo

4. Others (Pls specify)

7. Religion

1. Christainity

2. Islam

3. Others (Pls specify) ---------------------------------

8. Nameof the University or Polythechnic where you graduated --------------------------------------------------------------------------------------------------------------------------------------------------------------------------------------

9. Grade in School

1. First class

2. Second class

3. Third class

4. Pass

10. Course of study

1. Arts

2. Basic Sciences

3. Social Sciences

4. Medical Sciences

11. Do you smoke?

1. Yes

2. No

12. Do you drink?

1.Yes

2. No

SECTION B

13. Have you had Malaria in the last

1. 3 months

2. 6 months

3. 1 year

4. > 1 year

14. How many times do you usually have Malaria in a year --------------------------------------------------------

15. How many times in the last 5 years have you been admitted into the hospital because of Malaria (Pls specify) -----------------------------------------------------------------

16. What causes Malaria (Pls specify) -------------------------------------------------------------------------------------------------------------------------------------------------------------------------------------------------------------------------

17. Mention the symptoms of Malaria that you know? ------------------------------------------------------------------------------------------------------------------------------------------------------------------------------------------------------------------------------------------------------------------------------------------------------------------------------------------

18. How do you treat Malaria when you have it?

1. Buy drugs prescribed by self

2. Visited a health centre/doctor

3. Used drugs suggested by fiends

4. Went to patent medicine store

5. Visited an herbalist/native doctor

19. How long after onset of symptoms do you commence treatment (Pls specify)---------------------------

20. Which antimalaria did you use first when you had Malaria (Pls specify)--- ------------------------

21. Do you take chloroquine to cure your malaria? (Pls specify)---------------------------

22. When do you usually have Malaria?

1.Dry Season

2. Wet Season

23. How do you prevent yourself from having Malaria? (Pls specify)----------------------------

-----------------------------------------------------------------------------------------------------------------------------------------------------

24. Do you use any prophylaxis against Malaria?

1. Yes

2. No

25 .Have you ever heard of Insecticide treated net before?

1. Yes

2. No

26. Have you ever seen Insecticide treated net before?

1. Yes

2. No

27. Have you ever used Insecticide treated net before?

1. Yes

2. No

28. Are you currently using Insecticide treated net?

1. Yes

2. No

29. How long have you been using Insecticide treated net?

1. Yes

2. No

30. The price of insecticide treated net in my own opinion is

1. Too expensive

2. Expensive

3. Fair enough

4. Cheap

31. How frequently do you reapply the chemicals or treat your nets? (Pls specify) ----------------------------------------------------------------------------------------------------------------------------------------------------------------

32. What type of ITN do you use? (Pls specify) --------------------------------------------------------------------------------------------------------------------------------------

33. Since you have started using the ITN how has it affected the frequency of Malaria attack?

1. No longer have Malaria

2. Much reduced

3. Slight reduction

4. The same frequency

5. Increased frequency of Malaria

34. Do you know how ITN protects?

1. Yes

2. No

35. How do you get to know about ITN?

1. Friends/Relatives

2. School

3. Media

4. Hospital

5. Others (Pls specify) -------------------------------------------------------------------------------------------------------------------------------------------------------------

Thank you for your attention!!!!!!!
